# Metagenomic analysis of bloodstream infections in patients with acute leukemia and therapy-induced neutropenia

**DOI:** 10.1038/srep23532

**Published:** 2016-03-21

**Authors:** P. Gyarmati, C. Kjellander, C. Aust, Y. Song, L. Öhrmalm, C. G. Giske

**Affiliations:** 1 Karolinska Institutet, Department of Laboratory Medicine, Alfred Nobels Allé 8, Stockholm, 17177 Sweden; 2 Karolinska University Hospital, Department of Clinical Microbiology L2:02, Stockholm, 17176 Sweden; 3 Karolinska Institutet, Department of Medicine, Division of Hematology, Stockholm, 17176 Sweden; 4 Karolinska Institutet, Department of Medicine, Solna, Infectious Diseases Unit, Center for Molecular Medicine, Karolinska University Hospital, Stockholm, 17176 Sweden; 5 Royal Institute of Technology, Science for Life Laboratory, Stockholm, 17176 Sweden

## Abstract

Leukemic patients are often immunocompromised due to underlying conditions, comorbidities and the effects of chemotherapy, and thus at risk for developing systemic infections. Bloodstream infection (BSI) is a severe complication in neutropenic patients, and is associated with increased mortality. BSI is routinely diagnosed with blood culture, which only detects culturable pathogens. We analyzed 27 blood samples from 9 patients with acute leukemia and suspected BSI at different time points of their antimicrobial treatment using shotgun metagenomics sequencing in order to detect unculturable and non-bacterial pathogens. Our findings confirm the presence of bacterial, fungal and viral pathogens alongside antimicrobial resistance genes. Decreased white blood cell (WBC) counts were associated with the presence of microbial DNA, and was inversely proportional to the number of sequencing reads. This study could indicate the use of high-throughput sequencing for personalized antimicrobial treatments in BSIs.

Systemic infections, such as BSIs can develop in hematological malignancies due to inherent immune defects and therapy-related immunosuppression, and are associated with increased mortality^1^. BSIs are routinely diagnosed by blood culture and treated by empirical broad-spectrum antimicrobials. Antimicrobial treatment might be inappropriate due to the lack of coverage of the underlying pathogen(s), or antimicrobial resistance of the causative pathogens^2^. Blood culture requires relatively large sample volumes, only detects culturable pathogens and represents a narrow spectrum of the microbes present in a sample^3^. Furthermore, only 10–30% of blood cultures from febrile neutropenia, and 50% of blood cultures from septic shock are positive^1^,^4^,^5^. Although bacteria are the most commonly detected pathogens, fungal and viral infections also represent major complications in hematological malignancies^4^,^6^,^7^. *Candida* and *Aspergillus* species are the most commonly occurring fungal pathogens, but others, including *Fusarium* and *Trichosporon*, can also be detected^8^.

With the recent advantages of high-throughput sequencing technologies, it has become possible to reconstruct the taxonomic diversity of uncultured microbial communities, even in complex clinical samples. The two main approaches remain the amplicon sequencing (mostly restricted to the 16S rDNA universal gene in bacteria) and shotgun metagenomics, which allows untargeted sequencing of DNA in a given sample. Compared to the 16S rDNA sequencing approach, shotgun metagenomics not only allows the analysis of bacterial diversity in clinical samples, but can also provide information on the presence of parasitic, fungal and viral pathogens. An important clinical aspect is microbial resistance: sequencing of the 16S rDNA gene does not provide information regarding resistance patterns, while shotgun sequencing can identify resistance genes, their point mutations and also chromosomal resistance mechanisms. The latter approach also permits functional annotation of the sequencing data by assigning molecular functions to sequencing reads.

The aim of this study was to characterize the microbial content of blood samples from neutropenic patients with newly diagnosed acute leukemia. Sampling was performed at different time points before and after the initiation of antimicrobial treatments, and sequence analysis was combined with functional annotation.

## Results

### Sequencing reads

**Figure 1:**
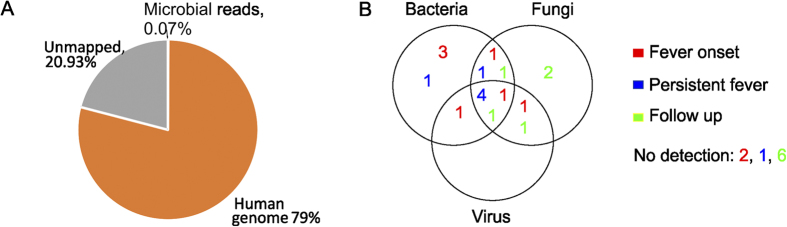
(**A**) Distribution of sequencing reads between human and microbial origins. (**B**) Distribution of the detected microbes. Colored numbers indicate the number of occurrences per sampling category (red numbers: fever onset, blue: persistent fever, green: follow up). Two fever samples did not contain microorganisms, and there were no detections of pathogen DNA in 1 persistent fever sample and 6 follow up samples.

In total, 1,005,260,502 reads were generated (excluding spike in samples), with 33.5 million reads being sequenced per sample on average. Seventy-nine percent of the reads were mapped to the human genome, while 20.93% of the reads were unmapped. Microbial reads consisted 0.07% of the total reads (Fig. 1). As shown in previous studies, human DNA exceeds microbial DNA approximately 10^7^–10^8^ –fold in sepsis^9^,^10^. The applied background suppression treatments reduced human DNA approximately 10,000-fold as sequencing reads aligned to the human genome outweighed microbial reads by 10^4^ on average.

### Microbial content of blood samples per sample category

Samples from nine patients were included in this study; in two patients, no microbial detection occurred. Samples (n = 27) were analyzed at different time points during antimicrobial treatments: 1) at inclusion (n = 1), 2) at fever onset (before antibiotic treatment started, n = 9), 3) 1 day after antibiotics were given (n = 7), and 4) follow up (1–5 days after the start of antibiotic treatment started, n = 11).

Three out of nine fever onset samples only contained bacteria, while four out of seven persistent fever samples contained bacteria, fungi and viruses (Fig. 1B).

**Figure 2:**
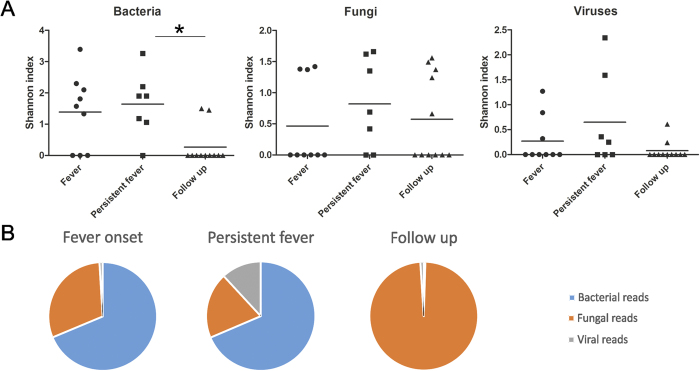
(**A**) Shannon’s diversity index is shown for bacteria, fungi and viruses at different time points of neutropenic fever. Significant decrease can be seen in bacterial diversity in follow up samples, due to antibiotic treatment. *denotes p ≤ 0.05. (**B**) Distribution of microbial reads per time points.

Bacterial reads were detected mostly at fever onset and in persistent fever (69% of all reads in both categories), but in less than 1% in follow up samples. The diversity of bacterial taxa did not change during fever, but showed a significant decrease in follow up samples, likely due to antibiotic treatment (Fig. 2A). Viral reads were dominantly detected during persisting fever (Fig. 2), and mostly consisted of bacteriophages. The diversity of fungal and viral taxa did not show significant changes between disease states.

### Distribution of bacterial taxa in different time points of sampling

**Figure 3:**
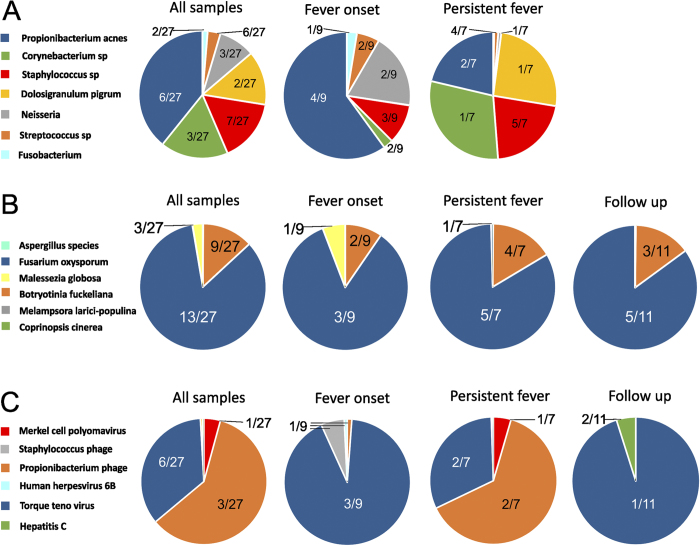
(**A**) Distributions of bacterial reads are shown in different diseases stages, with *Propionibacterium* dominating during the initial fever episode, while *Corynebacterium, Staphylococcus and Dolosigranulum* were also detected in persisting fevers. Bacterial taxa which consisted of <1% of all reads in a sample are not shown. (**B**) Distribution of fungal taxa detected in the samples used in this study indicate the presence of *Fusarium* in different disease stages, possibly implying subclinical fungal infections. (**C**) Distribution of reads belonging viral taxa at different sampling time points. Numbers within the pie charts indicate the number of samples positive for the given pathogen.

The bacterial content of the samples was dominated by *Propionibacterium acnes*, *Corynebacterium spp* and *Staphylococcus spp* in agreement with a 16S rDNA study on BSIs^3^. *Dolosigranulum pigrum* (in follow up samples) and *Neisseria spp* (during persistent fever) were also frequently detected (Fig. 3A).

The distribution of bacterial taxa per sample type revealed a difference between sampling time points: *P. acnes* was the dominant taxa only in fever onset samples, and was not detected in follow up samples at all. In samples with persisting fever, *Corynebacterium spp*, *D. pigrum* and *Staphylococcus spp* were dominant, while *S. spiritivorum* was the most prominent taxa in follow up samples.

### Distribution of fungal taxa in different time points of sampling

*Fusarium oxysporum*, a known fungal pathogen in immunocompromised patients, was detected most frequently in every sampling category (in 13 samples out of 27, Fig. 3B). Other detected fungal taxa also represent opportunistic infections (e.g., *Aspergillus spp* and *Malessezia globosa*) characteristic for immunocompromised patients.

### Distribution of viral taxa in different time points of sampling

Overall, phage related to *Propionibacterium* were the most dominant viral taxon (Fig. 3C), along with *Torque Teno Virus* (TTV). TTV was detected in 6 samples from 3 patients, while *Propionibacterium* phages were detected in 3 samples, from 3 patients. Amongst the known oncoviruses, *Merkel cell polyomavirus* and *Hepatitis C* were detected. Human endogenous retroviruses, as part of the human genome were removed from the analysis.

### Changes in microbial content in individual samples throughout the disease

As shown, antibiotic treatments were typically efficient against bacterial infections (e.g., *P. acnes* or *Neisseria spp.* in patients 1, 4, 5, 7), but less effective against species such as Pseudomonas (patient 2). Viruses and fungi were also often detected and were not affected by the antiviral/-fungal prophylaxis. When the infection was dominantly fungal or viral (patients 3, 5, 6), persistent fever samples contained the most reads, while in bacterial infections, fever onset samples had the highest number of reads.

**Figure 4:**
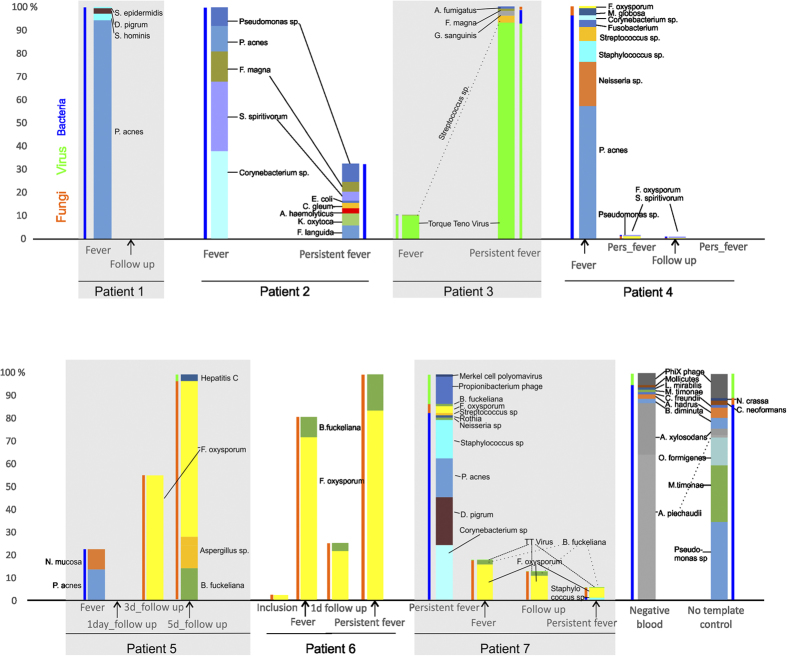
Relative abundances of bacterial, fungal and viral taxa are shown in individual samples. The highest bar for each patient represents the highest number of reads; the heights of other bars of the same patient are proportional to the highest. Wider bars represent taxa on the species level, slim bars show domains. Only taxa with ≥1% relative abundance are shown.

In patients 3 and 6, fungal and viral taxa were dominant, and the number of reads increased with fever (Fig. 4).

### Laboratory data

**Figure 5:**
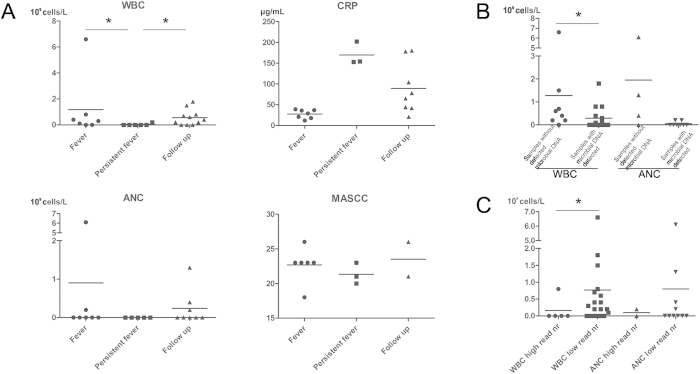
(**A**) Clinical characteristics of the samples used in this study, measured at the time of sampling. (**B**) Comparison of white blood cell count and absolute neutrophil count between samples with and without the presence of microbial DNA. (**C**) Comparison of WBC and ANC between samples with the highest microbial reads (>10,000) versus samples with microbial reads lower than 10,000. *denotes p ≤ 0.05. WBC: white blood cell count, ANC: absolute neutrophil count, CRP: C-reactive protein, MASCC: Multinational Association for Supportive Care in Cancer risk score.

WBC and ANC were compared between patients in different disease stages (Fig. 5A), and with and without detected microbial DNA. WBC was significantly lower in patients when microbial DNA was identified (p ≤ 0.05, Fig. 5B).

Samples with the highest microbial reads (>10,000 reads, n = 6) had significantly lower WBC (p ≤ 0.05) and lower ANC (p ≥ 0.05) than samples with less microbial reads found (Fig. 5C).

### Bacterial resistance

Tetracycline resistance genes were found in 2 samples (patient 4, fever sample, and patient 7, persistent fever sample 1). MLS (Macrolide Lincosamide Streptogramin) resistance genes were found in 4 samples (patient 3 (persistent fever), patient 4 (fever and persistent fever samples), and patient 7 (persistent fever sample 1)).

### Gene ontology (GO) analysis

In bacterial reads, significant differences were found in molecular processes (binding processes and hydrolase activity) between persistent fever and follow up samples (p ≤ 0.05, Fig. S1a). Metabolic processes also showed a difference between these two groups (p ≤ 0.05, Fig. S1b).

In fungal reads, molecular functions and biological processes did not show changes throughout the disease, assuming the lack of active antifungal drugs (Fig. S2).

The molecular functions and biological processes did not show a significant change in viral reads, although the hydrolase activity was elevated in persistent fever samples (p ≥ 0.05, Fig. S3).

## Discussion

Although the blood is considered sterile in physiological state, it may harbor dormant microbes, or microbes can enter the bloodstream due to translocation or pathological conditions^11^. BSI is treated with empirical broad-spectrum antimicrobials, which are often not efficient against the invading microbes due to the lack of specificity or resistance mechanisms^2^. Even though detection of microbial DNA in the blood does not necessarily imply the presence of viable pathogens, it is associated with worse clinical outcomes^5^,^12^,^13^; Fig. 5 in present study).

In this study, blood samples from patients with acute leukemia and suspected bloodstream infection were subjected to characterizing their microbiota using shotgun metagenomics. To our knowledge, this is the first investigation of uncultured microbial content and their resistance genes in blood samples from BSIs. Our results corroborate previous findings that the routinely used blood culture only detects a proportion of pathogens^3^, and our findings confirm the presence of viral and fungal pathogens in immunocompromised patients^7^,^8^.

The risk of infection is much greater in leukemic patients due to comorbidities and side effects of chemotherapy. BSI is often caused by bacteria, but can also be caused by fungi and viruses^7^,^8^. While bacterial infections are diagnosed with blood culture and treated with antibiotics, fungal and viral infections often go undetected in BSI cases, although they are associated with higher mortality rates in cancer patients^8^,^14^. As expected, the employed antibiotic treatment was effective against the majority of bacteria, but dominantly viral (including bacteriophages) and fungal pathogens were detected in 4 patients (Fig. 4). Fungal infections are an important cause of mortality in neutropenic cancer patients^8^,^15^, with A. fumigatus and C. albicans being mainly responsible for the infections, along with Fusarium and Trichosporon species. Posaconazole, which was used as fungal prophylactic is effective against most fungal pathogens but resistance can occur against this drug in Fusarium species^15^,^16^,^17^,^18^. Fusarium oxysporum was the most commonly detected fungal species in the current study (patients 5–7, Fig. 3B) with fungal load changing with fever (Fig. 4), which might indicate low level of persistent, possibly subclinical fungemia.

Viruses commonly occur in leukemic patients with febrile neutropenia^6^,^7^. For the patients included in this study, acyclovir was used as a prophylactic drug to prevent Herpesvirus-related infections. Herpesviruses are commonly detected in leukemic patients with neutropenia^6^,^7^, and were detected in 2 samples from 1 patient in our study as well, while TTV was the most commonly detected viral species (3 patients, 6 samples). TTV has been detected in leukemic patients but its causative role has not been proven; it was hypothesized however that TTV can be a co-virus in participating in the disease etiology in leukemia^19^. Bacteriophages are considered as part of the normal microbiota^20^; in this study, phages have been detected dominantly during persisting fever directly after antibiotic treatment, indicating that the origin of phages were bacteria degraded by antibiotics (Figs 2 and 3C). Additionally, antibiotic treatment is a strong inducer of the release of bacteriophages^21^,^22^, which can explain their elevated hydrolase activity (Fig. S3). In case of detection of bacteriophages, their corresponding host bacteria were also detected as phages exert high level of host specificity^23^.

Bacteria mostly occurred in fever and persistent fever samples, but were almost non-existent in follow up samples, due to the antibiotic treatment (Fig. 2). *P. acnes, Staphylococcus and Corynebacterium spp* were detected commonly in samples included in this study (Fig. 3A). *Dolosigranulum pigrum* and *Neisseria spp* bloodstream infections were also frequently detected, as they can be opportunistic pathogens in patients with immunosuppression^24^,^25^,^26^,^27^. Many bacteria have been detected and/or have been implied as causative in cancers^28^. Acinetobacter has been detected in acute leukemia and was postulated to play a role in bacteria-human cell lateral gene transfer^29^; this genus was detected in 5 samples from 2 patients in this study. Also, mucosal injury or damage in the lumen can also occur as a side effect of chemotherapy and opportunistic bacteria can enter the bloodstream in hematological malignancies^3^,^4^,^28^.

GO analysis of bacteria showed significant changes with antibiotic administration (Fig. S1), implying the effectiveness of antimicrobial drugs and adaptation of bacteria. GO analysis also confers the notion of resistance of the F. oxysporum strains as there were no changes in molecular functions and biological processes throughout the treatment (Fig. S2) despite of antifungal prophylaxis. The elevated ratio of transport processes in fungal reads might indicate resistance to drugs as the rate of transport of antimicrobial agents can be insufficient compared to the efflux pumps, resulting in inefficient doses^30^. The elevation of hydrolase activity in viral reads (Fig. S3) is most likely due to the release of phages with the degradation of bacteria in persistent fever samples (1 day after AB administration), as no changes were observed between fever onset and follow up samples.

In summary, this work confirms the presence of viral and fungal pathogens alongside bacteria and antibiotic resistance genes in leukemic patients with neutropenic fever. The presence of microbial DNA and the number of sequencing reads were correlated with lower WBC counts in blood samples included in this study. Altogether, our results imply the possible utility of this technology in personalized medicine in the antimicrobial treatment of patients with acute leukemia.

## Materials and Methods

### Study population and sampling

Eight patients with acute myeloid leukemia and one patient with acute lymphocytic leukemia considered suitable for dose intensive antitumoural treatment at the Hematology Center, Karolinska University Hospital in Stockholm, Sweden, were enrolled for this study upon diagnosis. Included patients were sampled with two 4.5 mL EDTA tubes for venous blood at different time points: 1) at hematological diagnosis, 2) at fever onset during neutropenia before intravenous broad-spectrum antibiotic treatment was initiated, 3) persisting fever during intravenous broad-spectrum antibiotic treatment, and 4) follow-up samples 1–5 days after the initiation of antibiotic treatment.

Data on white blood cell count (WBC), absolute neutrophil count (ANC), C-reactive protein (CRP) levels, Multinational Association for Supportive Care in Cancer (MASCC) Risk Scores, and hematological diagnoses were extracted retrospectively from the patients’ medical records. Samples were handled anonymously.

### Antimicrobial treatments

All patients were treated with empirical broad-spectrum antibiotics at fever onset, according to international IDSA guidelines. Five of the patients received ciprofloxacin prophylaxis. Acyclovir and posaconazole were used as antiviral and antifungal prophylactics for all patients, in accordance with the local hospital guidelines (Table S1).

### Ethics statement

All subjects provided written, informed consent. The study, and all experimental protocols used in this study was approved by The Regional Ethical Review Board, Stockholm (Regionala Etikprövningsnämnden Stockholm, recordal 2012/1929-31/1), and were carried out in accordance with the approved guidelines.

### Definitions

Fever was defined as a single oral temperature of ≥38.5 °C or a temperature of >38.0 °C persisting for >1 hour. Neutropenia was defined as a neutrophil count of ≤0.5 × 10^9^ cells/L, or a higher count with an expected decrease to ≤0.5 × 10^9^ cells/L within 24 hours.

### Sample preparation and sequencing

Blood samples for sequencing were drawn into sterile 4.5 ml Vacutainer (Becton Dickinson, Franklin Lakes, NJ USA) tubes, were kept at 4 °C and processed to DNA extraction within 1–24 hrs. MolYsis Complete5 kit (Molzym Life Science, Bremen, Germany) was used to extract bacterial DNA following the manufacturer’s instructions with the following exceptions: 5 minutes were used for the final elution instead of 1, and samples were dissolved in 50 μl water instead of 100 μl. One μg of the eluted DNA was then processed to NebNext microbiome enrichment (New England Biolabs, Ipswich, MA, USA) following the manufacturer’s instructions. Ten ng DNA was subjected to multiplex displacement amplification, using the GenomiPhi V2 DNA amplification kit (GE Healthcare, Little Chalfont, United Kingdom), with 90 minutes amplification resulting in 2–4 μg DNA. Two μg DNA was used for library preparation with the Nextera XT kit, and libraries were processed to a 2*100 base pair PE sequencing on a HiSeq 2500 instrument. The data generated in this study was uploaded to the NCBI Sequencing Read Archives under experiment SRX1381258.

### Validation of the assay

*Escherichia coli* NCTC 9001 genomic DNA was spiked in to microfiltered human blood (Sigma-Aldrich) in 10-fold dilutions from 1 million CFU to 0. No template controls did not generate sufficient background amplification with the standard protocol for sequencing, therefore the MDA reaction was performed for 3 hours and processed as described above. *E. coli* DNA spiked into sterile blood was detected down to 10 CFU. Control samples (microfiltered human blood, “Negative blood”) and a sample without added DNA (“No template control”, Fig. 4) were dominated by bacterial species, *Achromobacter piechaudii* and *xylosodan*s, *Pseudomonas* species and *Massilia timonae*, with several of them have already been reported as reagent contaminants^31^.

### Data analysis

Reads shorter than 30 bp and with Phred quality scores below 30 were discarded using the Fastx toolkit. Paired end reads were merged using the Flash software^32^, while unpaired reads were discarded. RTG Core 3.4^33^ was used to filter reads against the human genome (build Hg_19), and to map the unmapped reads to fungal, bacterial and viral whole genome sequences (downloaded from the NCBI Microbial Genomes Resources (http://www.ncbi.nlm.nih.gov/genomes/MICROBES/microbial_taxtree.html)), with minimum *e* value set to 10^−6^. Duplicate reads were removed from the analysis using RTG Core 3.4. Taxa detected in the no template control sample, or in the spike in sample with 0 copies of *E. coli*, or with ≤10 reads/taxon were not included. The ARG-ANNOT database was used to detect antibiotic resistance genes. GO analysis was performed using the Blast2GO v3 software, with *e* = 10^−6^. Statistical analysis was performed using the Mann-Whitney U-test, with significance set to 0.05.

## Additional Information

**How to cite this article**: Gyarmati, P. *et al.* Metagenomic analysis of bloodstream infections in patients with acute leukemia and therapy-induced neutropenia. *Sci. Rep.* **6**, 23532; doi: 10.1038/srep23532 (2016).

## Supplementary Material

Supplementary Information
