# Supplementary material for: Metagenomic analysis of bloodstream infections in patients with acute leukemia and therapy-induced neutropenia
Source: Sci Rep. 2016 Mar 21;6:23532. doi: 10.1038/srep23532 (PMC4800731; doi:10.1038/srep23532)
Supplement: Supplementary Information [file srep23532-s1.pdf]

Metagenomics analysis of bloodstream infections in patients with acute leukemia and therapy-induced neutropenia

Gyarmati P, Kjellander C, Aust C, Song Y, Öhrmalm L, Giske CG

Supporting Information

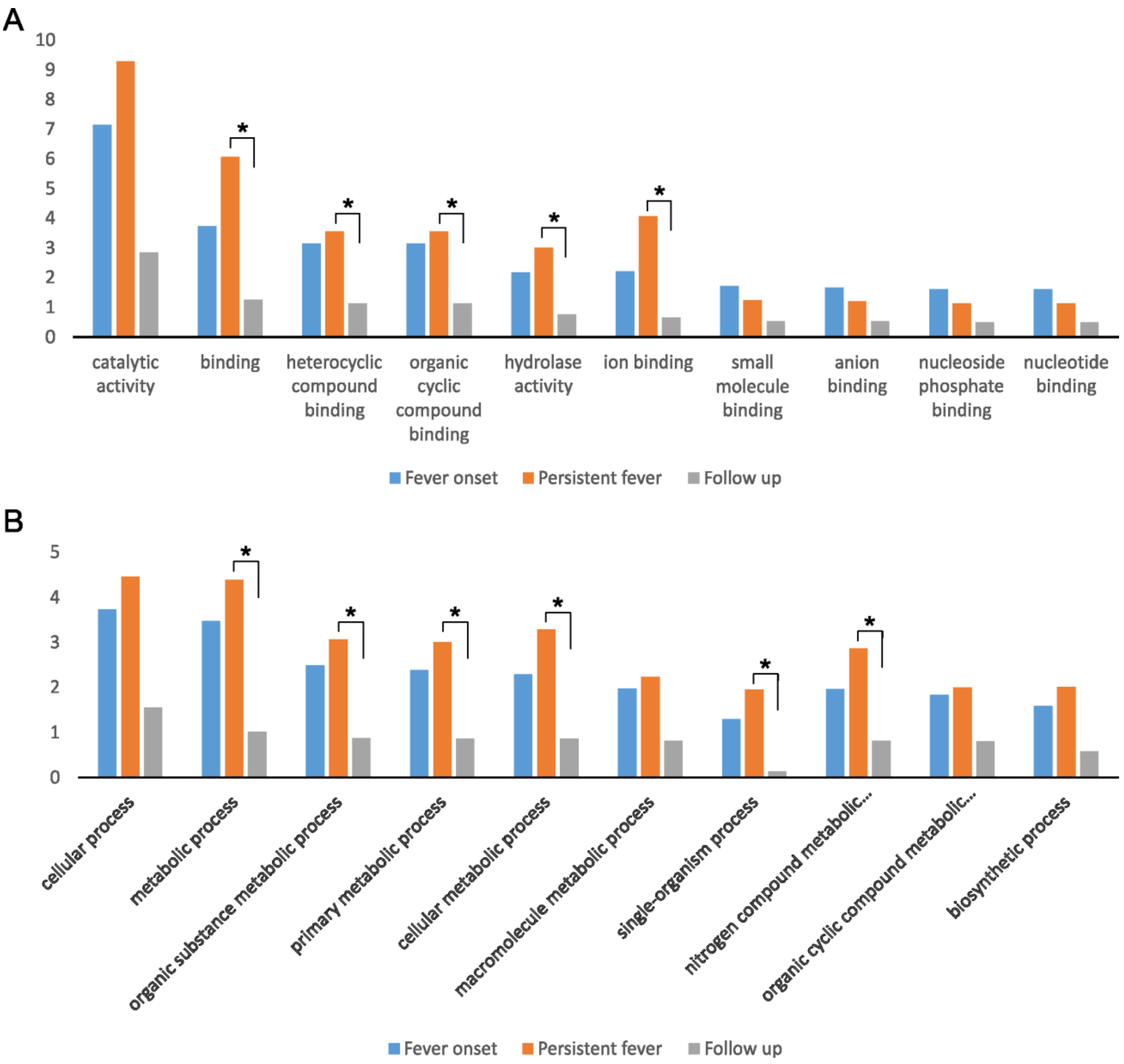

Fig. S1 (A) Relative abundance of molecular functions in bacterial reads based on the gene ontology analysis. Ten processes with the highest abundances in fever onset samples are shown. (B) Relative abundance of biological processes in bacterial reads based on the gene ontology analysis. Ten processes with the highest abundances in fever onset samples are shown.

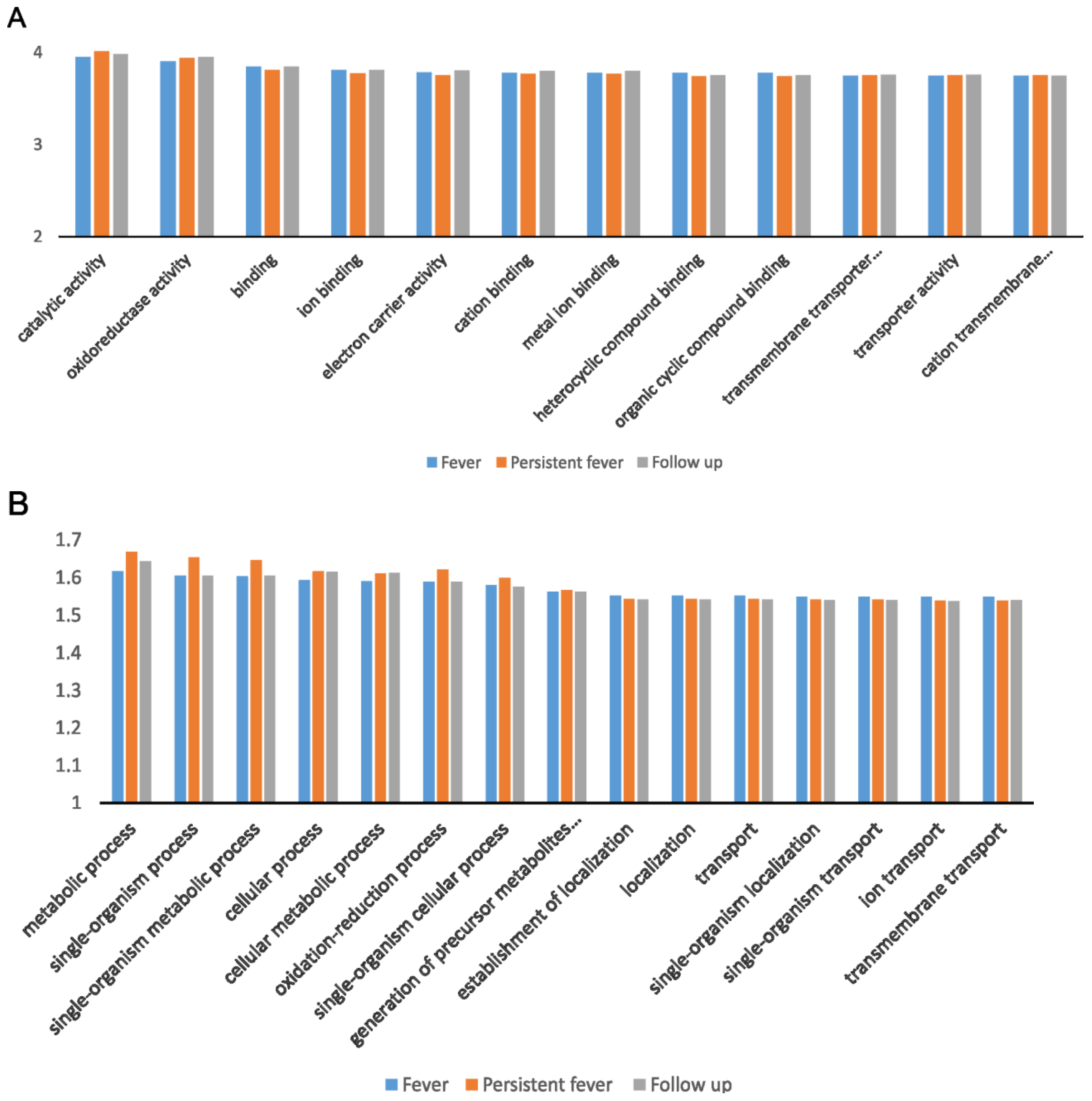

Fig. S2 (A) Relative abundance of molecular functions in fungal reads based on the gene ontology analysis. Ten processes with the highest abundances in fever onset samples are shown. (B) Relative abundance of biological processes in fungal reads based on the gene ontology analysis. Ten processes with the highest abundances in fever onset samples are shown.

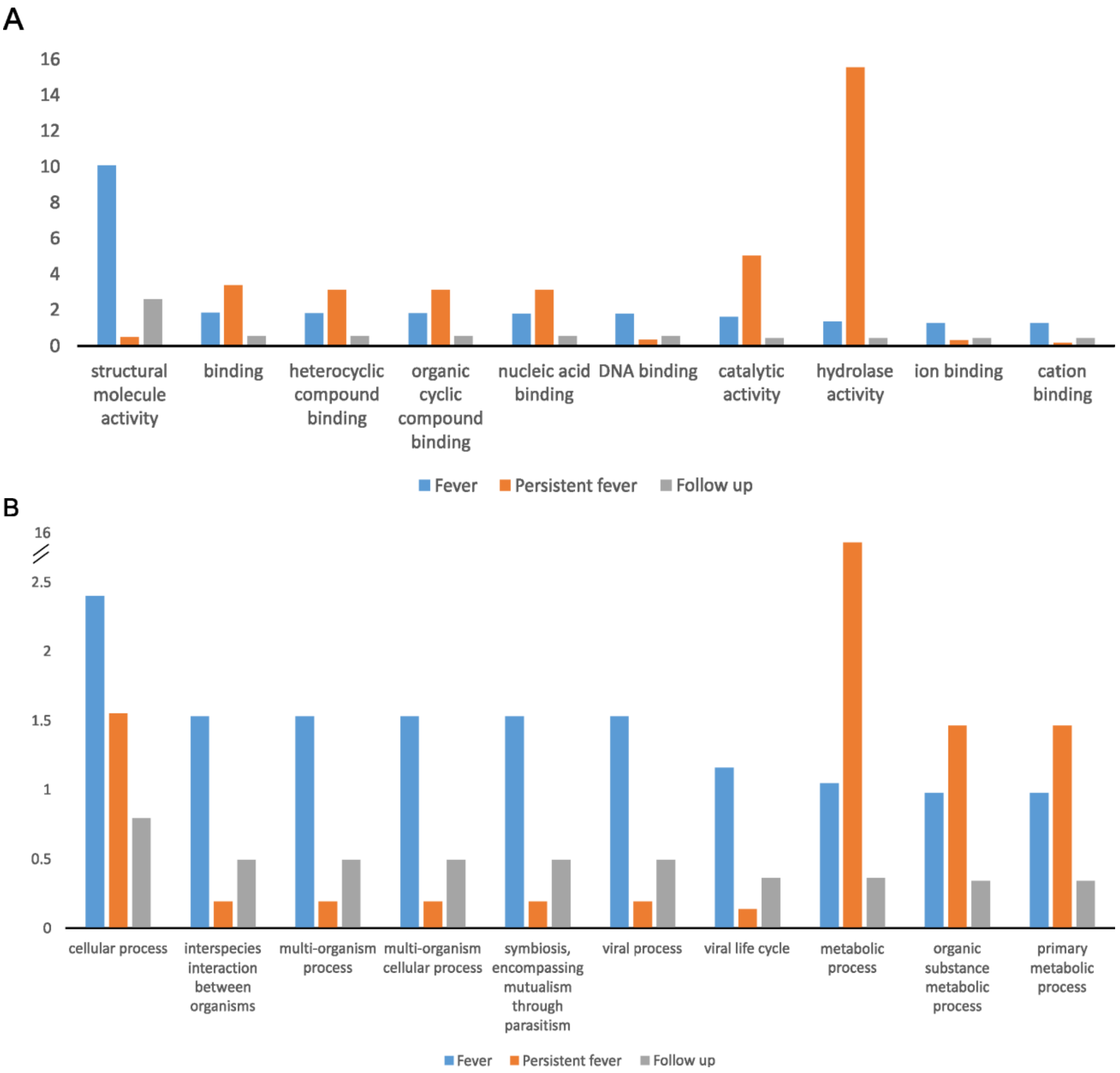

Fig. S3 (A) Relative abundance of molecular functions in viral reads based on the gene ontology analysis. Ten processes with the highest abundances in fever onset samples are shown. (B) Relative abundance of biological processes in viral reads based on the gene ontology analysis. Ten processes with the highest abundances in fever onset samples are shown.

	Age	Gender	Chemotherapy		Fever	Blood culture result	WBC	ANC	CRP	Broad-spectrum antibiotics	Antibiotic prophylaxis	Antiviral prophylaxis	Antifungal prophylaxis	MASCC	Hematological diagnosis
**Patient 1**	50	M	cytarabine + daunorubicin	Sample 1	x	Alpha Streptococcus	0.1	0	36	piperacillin/tazobactam	-	valacyclovir	posaconazole	18	acute myeloblastic leukaemia
				Sample 2	-	ND	0.2	ND	180	meropenem	-	valacyclovir	posaconazole	21	acute myeloblastic leukaemia
**Patient 2**	54	M	cytarabine + daunorubicin	Sample 1	x	Alpha Streptococcus	0	0	18	piperacillin/tazobactam	ciprofloxacin	valacyclovir	posaconazole	23	acute lymphoblastic leukaemia
				Sample 2	x	negative	0	0	ND	amikacin	-	valacyclovir	posaconazole	20	acute lymphoblastic leukaemia
**Patient 3**	65	F	cytarabine + daunorubicin	Sample 1	x	negative	0.3	0	29	meropenem	ciprofloxacin	valacyclovir	posaconazole	ND	acute myeloblastic leukaemia
				Sample 2	x	negative	0.2	ND	ND	meropenem	ciprofloxacin	valacyclovir	posaconazole	ND	acute myeloblastic leukaemia
**Patient 4**	25	M	cytarabine + daunorubicin	Sample 1	x	Alpha Streptococcus	0	0	12	piperacillin/tazobactam	ciprofloxacin	valacyclovir	posaconazole	ND	acute myeloblastic leukaemia
				Sample 2	x	Alpha Streptococcus	0	ND	ND	piperacillin/tazobactam	-	valacyclovir	posaconazole	ND	acute myeloblastic leukaemia
				Sample 3	-	negative	0	ND	178	piperacillin/tazobactam	-	valacyclovir	posaconazole	ND	acute myeloblastic leukaemia
				Sample 4	x	ND	0	ND	154	clindamycin	-	valacyclovir	posaconazole	21	acute myeloblastic leukaemia
**Patient 5**	51	M	cytarabine + daunorubicin	Sample 1	x	negative	0.4	ND	37	piperacillin/tazobactam	ciprofloxacin	valacyclovir	posaconazole	26	acute myeloblastic leukaemia
				Sample 2	-	ND	0.4	ND	44	piperacillin/tazobactam	-	valacyclovir	posaconazole	ND	acute myeloblastic leukaemia
				Sample 3	-	ND	0.8	0	ND	piperacillin/tazobactam	-	valacyclovir	posaconazole	ND	acute myeloblastic leukaemia
				Sample 4	-	ND	1.8	0.2	ND	piperacillin/tazobactam	-	valacyclovir	posaconazole	26	acute myeloblastic leukaemia
**Patient 6**	50	F	cytarabine + daunorubicin	Sample 1	-	ND	ND	ND	ND	piperacillin/tazobactam	-	valacyclovir	posaconazole	ND	acute myeloblastic leukaemia
				Sample 2	x	negative	0.8	0.2	ND	piperacillin/tazobactam	-	valacyclovir	posaconazole	23	acute myeloblastic leukaemia
				Sample 3	x	negative	ND	ND	ND	piperacillin/tazobactam	-	valacyclovir	posaconazole	ND	acute myeloblastic leukaemia
				Sample 4	-	ND	ND	ND	65	piperacillin/tazobactam	-	valacyclovir	posaconazole	ND	acute myeloblastic leukaemia
**Patient 7**	48	M	cytarabine + daunorubicin	Sample 1	x	negative	0	ND	153	piperacillin/tazobactam	-	valacyclovir	posaconazole	23	acute myeloblastic leukaemia
				Sample 2	x	Alpha Streptococcus	ND	ND	39	piperacillin/tazobactam	ciprofloxacin	valacyclovir	posaconazole	23	acute myeloblastic leukaemia
				Sample 3	-	ND	0	ND	78	piperacillin/tazobactam	-	valacyclovir	posaconazole	ND	acute myeloblastic leukaemia
				Sample 4	x	negative	0	ND	202	piperacillin/tazobactam	-	valacyclovir	posaconazole	ND	acute myeloblastic leukaemia

Table S1. Patients characteristics showing age, gender, chemotherapeutic treatment, blood culture findings, the applied broad-spectrum antibiotics, hematological diagnoses, antimicrobial prophylaxis and laboratory data. ND=no data; WBC=white blood cell count, 10^9 cells/L; ANC=absolute neutrophil count, 10^9 cells/L; CRP=C reactive protein, µg/ml; MASCC=Multinational Association for Supportive Care in Cancer risk score.
